# A Survey of the Techniques for The Identification and Classification of Human Actions from Visual Data

**DOI:** 10.3390/s18113979

**Published:** 2018-11-15

**Authors:** Shahela Saif, Samabia Tehseen, Sumaira Kausar

**Affiliations:** Computer Science Department, Bahria University, E-8 Islamabad 44000, Pakistan; stehseen.buic@bahria.edu.pk (S.T.); sumairakausar@bui.edu.pk (S.K.)

**Keywords:** computer vision, action recognition, visual action recognition, deep learning

## Abstract

Recognition of human actions form videos has been an active area of research because it has applications in various domains. The results of work in this field are used in video surveillance, automatic video labeling and human-computer interaction, among others. Any advancements in this field are tied to advances in the interrelated fields of object recognition, spatio- temporal video analysis and semantic segmentation. Activity recognition is a challenging task since it faces many problems such as occlusion, view point variation, background differences and clutter and illumination variations. Scientific achievements in the field have been numerous and rapid as the applications are far reaching. In this survey, we cover the growth of the field from the earliest solutions, where handcrafted features were used, to later deep learning approaches that use millions of images and videos to learn features automatically. By this discussion, we intend to highlight the major breakthroughs and the directions the future research might take while benefiting from the state-of-the-art methods.

## 1. Introduction

Activity recognition involves an understanding of human actions. A human action is harder to define than to understand, and many attempts have been made in the literature to define it in one way or the other. Turaga et al. [1] provided an intuitive definition of an action as “simple motion patterns usually executed by a single person and typically lasting for a very short duration (order of tens of seconds)”. Moeslund and Graum [2] and Poppe [3] have defined action as “an atomic movement that can be described at limb level.”; whereas the activity can be considered a sequence of actions that can involve interactions among humans or between humans and the environment.

The recognition of human actions form videos is a challenging task. It requires work in multiple disciplines to be effectively executed and combined such as object recognition, background and foreground processing, semantic segmentation and human dynamics. There are two major types of recognition systems: one that involves the use of wearable sensors or associated devices and the other that uses cameras and wireless radio frequency modules. Among the first kind, a few approaches to action detection have involved the use of dedicated sensors such as mobile sensors [4,5,6] or physiological data [7,8]. Classifiers are used on these data for action recognition. These approaches promise a higher accuracy, but work in a limited domain. In the second approach, features are extracted from visual input including single object’s features such as position, shape, color or global features such as region occupancy or positional variations. Normal activity templates and abnormal activity templates are created that can be subjected to recognition through template matching methods or state space [9]. In recent years, there has been a significant increase in the uses of multi-modal video devices such as Kinect, which provides depth information apart from the color information from (regular) video cameras. Such systems can provide an accurate representation of a human shape, which is utilized to form various activity shape features [10]. Researchers have used kinematic joints [11], human posture [12] and even histogram-based approaches [13] for action recognition using such devices [14]. Since our focus is on devices that use traditional video data that do not include depth information, we shall not discuss these any further in the current study.

Video analysis has been performed at various levels of detail depending on the information we require from them. The few significant ones were given in a study by [15]:

1. Object scope understanding where only the positions of persons and objects are detected.
2. Tracking scope understanding where the trajectories and correspondence of objects are analyzed.
3. Pose-level understanding that involves the analysis of the position of human body parts.
4. Analysis of human activities and events.

There are several existing surveys that have explored the techniques for activity recognition. Some of these have divided these recognition approaches into single-layered and hierarchical approaches, as in the works of Aggarwal and Ryoo [15] and Cheng et al. [16]; while others like Moeslund et al. [2] and Poppe [3] have divided the work on the basis of action and activity. Aggarwal and Cai [17] have performed another survey on the same domain in which they reviewed the literature from three perspectives: (1) motion analysis with regards to body parts, (2) tracking from single or multiple camera perspectives and (3) using images for recognizing activities. Gavrila [18] also discussed action recognition techniques based on whole-body or hand motion tracking while discussing both 2D and 3D approaches.

**Figure 1:**
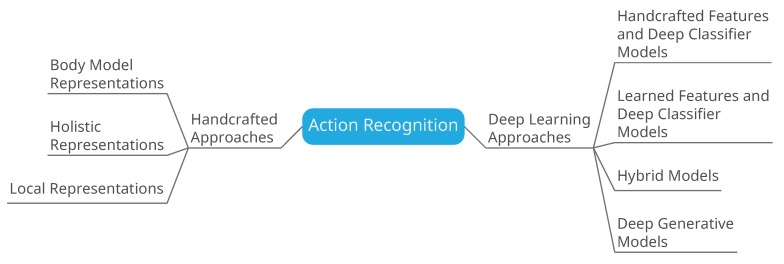
Classification of action recognition based on techniques employed for identification and classification of actions.

**Figure 2:**
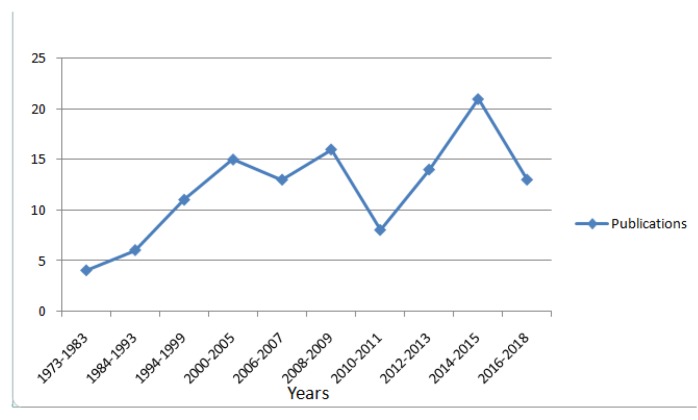
Research publications per year as discussed in the current study.

Handcrafted feature extraction techniques paired with classifiers have been used for action recognition for quite some time and with considerable success [19]. However, the availability of large amounts of data has made possible the use of deep networks for the task of action recognition [20]. The success of deep networks and in particular CNN is evident from the results on ImageNet [21]. A mention of the other studies that cover action recognition is provided in Table 1. This survey is oriented in a manner to review both the handcrafted recognition techniques and the deep learning techniques, as given in Figure 1. We also explore the effect of using local features for action recognition. Figure 2 shows the research interest in action recognition over the years. With time, the handcrafted approaches matured and started producing results that could be used in building real-time applications. The renewed interest came with the arrival of deep architectures in 2012 and later. There are many studies that have explored the applicability of deep architectures to activity recognition, both in conjunction with handcrafted approaches and in standalone capacity.

The rest of the paper is organized as follows: In the next section, we discuss some of the challenges of action recognition using data from videos. Section 3 gives an overview of the handcrafted approaches that essentially use handcrafted methods for identification of action in conjunction with a classifier for action classification. In Section 4, we take a look at the approaches that use deep learning. The deep learning approaches include: (i) approaches that make use of handcrafted features for identification that are given to a deep network for fine-tuning and classification; (ii) approaches that use deep networks both for the task of feature extraction and classification; (iii) hybrid approaches; and (iv) deep generative models. A critical discussion of the approaches follows the details of the datasets prior to the conclusion.

## 2. Challenges

The activity recognition process involves quite a few challenges and constraints that need to be dealt with at the time of both feature extraction and classification. Poppe has listed some of the significant ones in [3]. These are:

1. Inter-class variations: Different people perform different actions in their own ways, which at times show very low resemblance to one another, e.g., walking methods may differ in stride length or speed.
2. Intra-class similarities: Actions belonging to different classes may appear similar such as jogging and running.
3. View point variations: The same action if observed from two independent viewpoints can appear to be different, and the data collected as a result may indicate separate classes.
4. Environment: Cluttered or complex backgrounds can make the task of identification of clear human shapes much more difficult.
5. Temporal variations: Temporal variations occur both in terms of action performance/completion and action observation.

All these issues are addressed explicitly by the action recognition approaches as and when they arise. However, depending on the datasets that are being used and the feature selection techniques employed, the impact of these constraints may vary. There is, thus, no single strategy that can be applied for any particular problem while using different action recognition techniques. In the subsequent sections, we provide a review of various action recognition techniques along with their shortcomings. The organization of the techniques is based on the time of introduction and the growing complexity of the presented techniques.

## 3. Handcrafted Approaches

The interest in human action recognition is not a recent one, and scientists and researchers have over time been utilizing various techniques for action identification. Using spatial information about the human pose, which is generated by extracting various image features, we can classify human pose based on the similarity of the pose to some action.

### 3.1. Body Models

**Figure 3:**
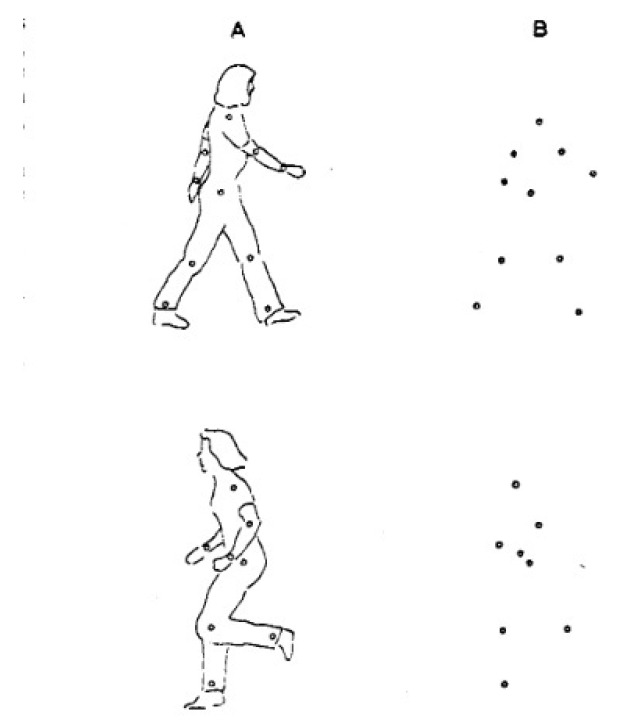
Moving light displays used for action recognition in [30].

Among the earliest attempts at action recognition, Johansson [30] used a simplistic representation of the human body that was comprised of readable light sources placed on joints (Moving Light Displays (MLDs)) and could determine the action based on the movement of joints. An example of these MLDs is given in Figure 3.

**Figure 4:**
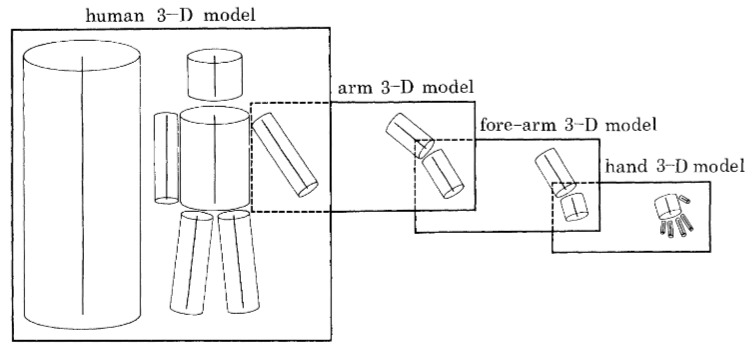
Human model created in 3D using 2D information in [31].

As a pioneer work in this field, these simplistic experiments paved the way for many more methods based on the same idea. The two predominant techniques that emerged as a consequence of this work are the (i) representation of motion as a 2D sequence of actions and (ii) generation of 3D models from 2D representations to recognize actions [24]. The variability of the human body’s shape poses many interesting challenges that have led researchers to construct 3D models of the human body. The earliest work along these lines was done by Marr and Nishihara [31], where they used cylindrical models for human body representation, as given in Figure 4.

Others have built on such models as well [32,33]; some have provided more flexible models using super quadrics [34] and textured spline models [35]. These models are difficult to compute and do not have the flexibility to provide solutions to problems such as view point variations, environment clutter or temporal variations. These worked in strictly controlled environments and were therefore soon replaced by improved techniques. They did, however, set the direction for future research for many years to come. The concepts of body models were picked up by researchers who used wearable devices and 3D data-collection devices such as Kinect for action recognition [4]. Such models have also provided accuracies up to 90%. These are not discussed in detail here, as the aim of the current study is ‘visual data’-only techniques.

### 3.2. Holistic Representations

Holistic representations do not require identification or marking of individual body parts unlike the body models discussed in the previous section. These approaches work by preprocessing the images by performing fundamental tasks such as background subtraction and feature extraction. Most techniques make use of contours and/or silhouettes of the human body [36,37,38].

Darell and Pentland [39] created a model where images of hand gestures were correlated with one another directly without the need to extract any features. However, for their work, they assumed a static black background, which may not be very practical. A significant work in the same direction was by Yamato et al. [40] in which they converted the time-sequential images into a unified image feature vector where only silhouettes were used. This feature vector is used as a symbol sequence that is evaluated using a Hidden Markov Model (HMM).

**Figure 5:**
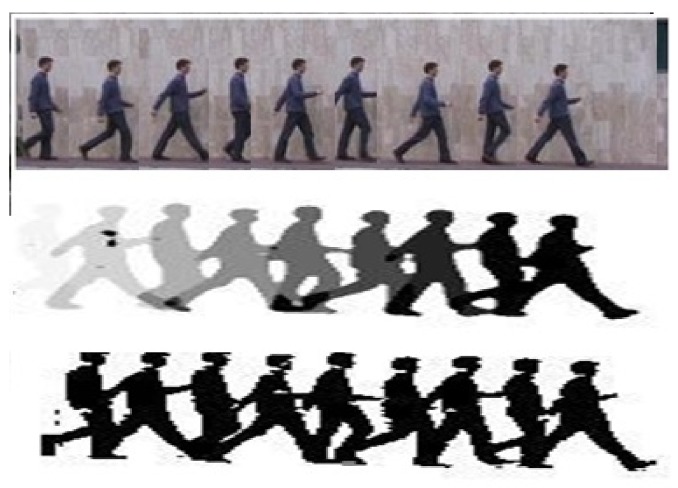
Top row: A walking sequence of a person; middle row: a Motion Energy Image (MEI) template; bottom row: a Motion History Image (MHI) template [41].

Work by Bobick and Davis [41] has had a tremendous effect on all future research on activity recognition. They created ‘Motion History Images (MHI)’ and ‘Motion Energy Images (MEI)’ from silhouettes that were integrated over the time domain (using frames’ information); see Figure 5 for a reference. MHI and MEI have been adapted and improved by many later works. Space-time volumes that a silhouette spans over in multiple frames were used in [42,43] as opposed to integrating the time-sequence into one image, as done by [41].

Elgammal et al. [44] and Weinland and Boyer [45] used chamfer distance to eliminate the affects of noisy silhouettes, which are caused due to cluttered backgrounds. Shape-context descriptors [46,47,48] were also used to the same effect. Silhouettes are insensitive to color, texture and context, but are not very effective in cases of self-occlusion. A better approach is the use of dense optical flows [49,50] and clustering these optical flows into motion blobs [51]. Optical flow fields were split into four different scalar fields by [52,53,54]. Optical flow fields do not require background subtraction, but are also sensitive to material properties, lightening, etc. Gradients are also used to extract image features [55]. Histograms of oriented gradients are used for object detection [56] and for action recognition [57]. Gradient features, like optical flows, do not require background subtraction, but are affected by material properties. Some studies have used optical flows in combination with gradients [58,59] and silhouettes [60] to achieve superior results.

The results of holistic representations are promising, but are incapable of handling viewpoint variations [51,61]. Improvements of these are local and deep approaches.

### 3.3. Local Representations

#### 3.3.1. Interest Point Detection

**Figure 6:**
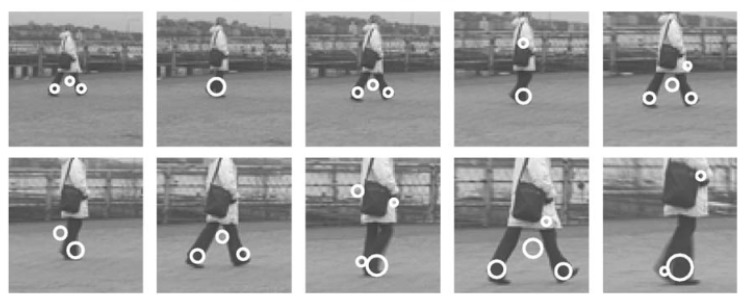
Spatio-temporal interest point detection for a walking person. Reprinted with permission from [62].

Work by Laptev [62] on space-time interest points paved the way for local representations for image feature extractions. The author adapted the Harris corner detector [63] to create a 3D-Harris detector that can detect spatial changes in orthogonal directions along with points that have large non-constant motion, as seen in Figure 6. The 3D-Hessian detector [64] uses second order derivatives instead of gradients as in the Harris detector for interest point detection.

#### 3.3.2. Local Descriptors

Earlier works in action recognition have used cuboid models for body representation [62,65], but were challenged by Messing [66] and Matikainen et al. [67] in terms of effectiveness and flexibility. An improvement on this is considered by using edge and motion descriptors.

##### Edge and Motion Descriptors

Histogram of oriented Gradients (HoGs) were used for motion detection by [68], and later, [56] extended this to the spatio-temporal domain, naming it HoG3D. Laptev [58] employed the same idea for optical flow fields, since they encode the pixel-level motion in videos, and created the Histogram of optical Flow (HoF). Dalal et al. [69] created a more robust version of HoF, the Motion Boundary Histogram (MBH). The calculation of optical flow fields is computationally expensive, and decompression techniques have been employed [70] to overcome this disadvantage.

#### 3.3.3. Trajectory-Based Approaches

One criticism to cuboid representations is that over a span of frames, the detected interest point may not lie at the same spatial location within the temporal bounds of a cuboid. Action trajectory is the tracking of a feature in the time domain. Trajectory-based action representations were widely adopted after the works of Messing et al. [66] and Matikainen et al. [67]. Wang et al. [71] integrated MBH, HoG and HoF to create a rich feature representation, where trajectories were calculated by using optical flow. Vig et al. [72], addressing the computational complexity of the prior technique, used ‘saliency-maps’ to extract the region of interest inside frames. In a similar approach, Jiang et al. [73] used local and global reference points along with trajectories to improve motion detection. Wang et al. [74] improved their original work by eliminating the effect of camera movements by using SURF and dense optical flows. The improved model was adopted by many, including Peng et al. [75], who have developed a multi-layer stacked Fisher Vector (FV) [76] with improved performance over the original model.

Handcrafted approaches are complex to build and hard to modify. These cannot be readily adapted to new or complex datasets, which has hindered their ability to provide a unified global solution. This was changed by the rapid increase in use of deep architectures for image analysis techniques. Given in the next section are various approaches based on deep learning; some of which also make use of handcrafted approaches in a limited capacity.

## 4. Deep Learning Approaches

With the advent of deep learning approaches that enable the learning of features along with the classification of them, we have seen the application of these in the field of action recognition with considerable success. In particular, convolutional neural networks have revolutionized the field of image classification and recognition [77,78,79,80] and are employed singularly or in conjunction with other architectures for action recognition tasks.

In general, we can categorize deep approaches into two major schemes based on network function: supervised approaches and unsupervised approaches. The supervised approaches include (i) networks that extract features from deep models and use other classifiers and (ii) networks that use deep models for end-to-end classification, as well as (iii) networks that use handcrafted features in conjunction with deep networks for classification [81]; while unsupervised and semi-supervised approaches are the deep generative models, such as autoencoders or adversarial networks.

The supervised approaches are split into three architectures or combinations and/or evolutions of these three architectures: Convolutional Neural Networks (CNN), Recurrent Neural Networks (RNN) and Long Short-Term Memory networks (LSTM).

CNN: CNN consist of a number of convolutional layers, each of which is responsible for feature extraction. The lower layers extract simple features, while the higher layers extract complex features by the use of filters at each layer. The filters are designed on the principle of weight sharing, which enables reducing the number of parameters to learn. Each layer that increases the depth and complexity of a network also inadvertently increases the dimensionality of the convolved features. CNN are used as effective feature learners, but their greatest strength is their ability to be used as end-to-end models for classification [82].

RNN: Recurrent neural networks have the ability to process feedback connections, which allows them to model sequential behavior. RNNs have found considerable success in handwriting recognition [83,84] and speech recognition [85,86], which led to their induction to modeling temporal associations among video frames to represent human action. The recurrent neural network effectively updates its current memory vector depending on three elements: current frame, previous memory vector and previous location of an object.

LSTM: Long short-term memory models are used in conjunction with various CNN and/or RNN models in order to represent long-term temporal dynamics and to do away with the vanishing gradient problem.

### 4.1. Handcrafted Features and Deep Classifiers

Handcrafted features have given promising results over the span of decades, where more and more sophisticated features emerged with time [71,72,73,74,75]. The appeal of using handcrafted features is to incorporate the time dimension of video sequences and to provide a ‘running start’ to a deep network. Kim et al. [87] proposed a modified convolutional neural network where the low level action information is represented by handcrafted features. The action sequence of any person in a video generates a 3D volume that is extracted using 3D Gabor filters [88]. These filters extract the outer boundary of an actor in a 2D or spatial plane, and when considered across multiple frames, they generate a spatio-temporal volume. These spatio-temporal volumes make the actions view-invariant. A 3D CNN is applied to each spatio-temporal volume, and features are extracted based on these. The features thus obtained are classified using a discriminative classification model [87]. Jhuang et al. [89] created a feed-forward hierarchical framework that detects spatio-temporal features of increasing complexity to measure ‘motion-direction sensitive units’. By taking the global max of each feature map containing scale- and position-invariant features [90,91,92], a feature vector is computed as a final representation. The approach is sensitive to the effectiveness of the handcrafted spatio-temporal feature detectors, which limits its effectiveness.

### 4.2. Learned Representations and Deep Classifiers

The three-dimensional convolutional neural networks aim to extract spatial features using the normal 2D transforms and employ the third dimension to extract temporal information [93]. The 3D convolutional network as presented by Ji et al. in [93] applies a 3D kernel, a spatial kernel extended in the time dimension by applying the same 2D filter to a particular spatial location in multiple frames. This makes the features obtained by 3D convolutions invariant to spatial translation with respect to time. The 3D convolutional neural network is shown to produce better results than its 2D counterparts [93]. Most 3D architectures constructed in this manner have a limit to the number of frames used for extracting information in the temporal domain, which makes them very rigid. However, a major restriction is the high computational cost and need for a large amount of trained data. Varol et al. [94] used longer temporal regions for performing 3D convolutions, and it was seen that extending temporal depth improves the performance of the network.

**Figure 7:**
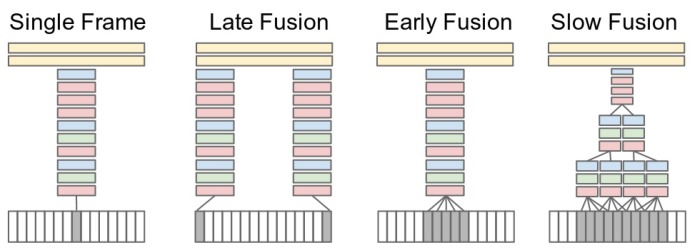
Fusion strategies for incorporating the temporal dimension in neural networks. Source: Reprinted with permission from [96].

Research has been focused on how to successfully incorporate the time dimension in deep networks. Ng et al. [95] have worked on the idea of temporal pooling and showed that max pooling provides the best results. Karpathy et al. [96] created different models for combining information from spatial and temporal domains; early fusion, late fusion and slow fusion; see Figure 7 for a reference. The single-frame approach uses one frame and applies a deep architecture over it without using temporal information. In late fusion, two images a certain number of frames apart are fed to two independent networks, fusing the results at the fully connected (FC) layers. Early fusion merges the frames at the pixel level before running them through a network. Slow fusion is an amalgam of late fusion and early fusion. The procedure requires the convolutional layers to be connected across multiple frames, thus providing the benefit of temporal convolution along with spatial convolution. Among the three, slow fusion performs better than the others because of its use of 3D convolutional kernels across multiple layers. Karpathy et al. have also experimented with multi-resolution models by creating a two-stream network. The ‘context’ stream processes a low resolution complete image, and a ‘fovea’ stream processes a high resolution cropped center of the image. The results of convolutions on both streams are combined at fully-connected layers to produce classification results. Using multi-resolution videos in separate, but identical networks reduces the number of parameters to learn and improves the accuracy [96]. Tran et al. [97] also used 3D CovNets while making use of a small 3 × 3 × 3 convolutional kernel throughout the network and showed that constant depth at every layer performed better than varying the temporal depth at each layer. This network, named C3D, gives rise to a generic descriptor, that averages the outputs of the fully-connected layers, with the aim of learning generic features from video, so that the network would not have to be fine-tuned for each independent task [97].

Using 3D filters increases the number of parameters and inevitably increases the cost and complexity of the network. Sun et al. [98] addressed this issue in their work and suggested factorizing a 3D filter into a 2D filter and a 1D filter. The benefit is reducing the number of network parameters from $n_{x}n_{y}n_{t}$ to $n_{x}n_{y}+n_{t}$, thus reducing the problem of kernel complexity by a factor of $O(n_{t})$. Others have exploited recurrent structures for achieving the same goals. Baccouche et al. [99] and Donahue et al. [100] have used a cascade of convolutional neural networks with Long-Short Term Memory (LSTM), where LSTMs are a class of recurrent networks [101]. In the work by Donahue et al. [100], the network named the Long-term Recurrent Convolutional Network (LRCN) performed an end-to end-training. The model has been successfully used not only for action recognition, but also for captioning of images and videos.

### 4.3. Hybrid Models

**Figure 8:**
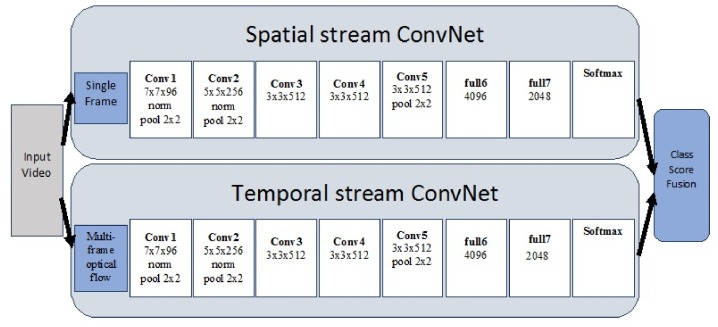
Two-stream architecture with the spatial stream using images and the temporal stream using optical flows. Source: [102].

Multi-stream models have been built on the idea of the separation between the spatial domain and temporal domain. Simonyan et al. [102] introduced this idea of multiple streams where they trained one convolutional network to extract spatial information about the video frames and another to capture temporal information using optical flows [103]. The two streams were later ‘fused’ using their softmax rates, as shown in Figure 8. They [102] have worked with layers of dense optical flows of consecutive frames, motion trajectories and bi-directional optical flows. The convolutional neural network is trained in a multi-task learning setting [104] by classifying both on the HMDB-51 and UCF-101 datasets and using two softmax layers, each of which computes a score on its respective dataset. Both streams are trained in the same manner, where the temporal network is an adaptation of the model by [105].Fiechtenhofer et al. [106] have shown improved results with a similar architecture that performs fusion at an intermediate layer.

Other works that have explored the idea of multi-stream networks are [107] in the form of a trajectory of pooled two-stream deep convolution descriptors. The network architecture is similar to that of [102], and they have used UCF-101 and HDMB-51 to compute multi-scale feature maps of the videos. Wang et al. [107] have further aggregated the computed dense trajectories over feature maps using the Fisher vector [108], but in terms of performance, this approach is no better than the original two-stream network.

### 4.4. Deep Generative Models

There is an ever-increasing amount of video data that are available over the Internet, but since most of this is consumer generated, thus they are not labeled. The potential of being able to use these data in an unsupervised environment to understand and predict the action sequences can give rise to endless possibilities. Generative models built for sequence analysis [79,109] have the ability to predict the next state of a sequence $x_{t+1}$ given a sequence of states $S=x_{1},x_{2},\ldots x_{t}$. Deep generative models do not require labels for training, but rely on finding accurate motion primitives [110,111,112]. Autoencoders have been used in research by many [113,114,115] for unsupervised learning of features through deep architectures. Yan et al. [116] captured video dynamics using a deep autoencoders, Dynencoder. The first layer of the model maps the input to hidden states; the second or prediction layer predicts the next hidden states based on the current ones, and the final layer is from the predicted hidden states to estimated input states. The training phase is followed by end-to-end fine-tuning.

Sirvastava et al. [109] created an LSTM autoencoder where two recurrent structures were used: encoder LSTM and decoder LSTM. The encoder LSTM receives input and learns compact representations, while the decoder LSTM uses these learned representations to reconstruct the input. An LSTM autoencoder can be used to predict the next states of a sequence, as well, and is thus more efficient than a 3D CNN. Another interesting approach is the use of adversarial networks [110]. In this work, two models were trained simultaneously; (i) a generative model that takes input data and generates a representation of them; and (ii) a discriminative model that tries to distinguish between real input and a generated representation. The harder it is for the discriminative model to differentiate between real and generated data, the better the learned representation and, thus, the model performance [110]. Mathieu et al. [117] have also used the adversarial model to train multi-scale CNN that avoid pooling layers. Their model is for video prediction, as well.

## 5. Datasets

The datasets for action recognition have evolved over time and have become more complex and realistic [20]. The earliest datasets such as KTH and Weizmann have a fixed number of subjects and a very limited number of action categories, as they were shot in controlled environments [118]. Datasets with increasing complexity include not only more action classes, but also complex backgrounds, multiple actors, occlusions and viewpoint variations; some even contain resolution inconsistencies [118]. A list of datasets used for action recognition is given in Table 2.

The most challenging datasets are the ones that involve YouTube videos and sports videos. These have the most variable backgrounds and viewpoint variations. Some YouTube videos are from user devices and have low camera stability and low resolution. A list of techniques and their accuracy is presented in Table 3 for further discussion. Nearly all of these papers have reported results on more than one dataset, but we have chosen to show only the ones that have reported the highest accuracy.

## 6. Discussion

It is interesting to see how the deep approaches in action recognition perform with respect to handcrafted or local approaches; since in terms of images, we have seen that deep architectures have outperformed the previous approaches by quite a wide margin [105]. An accurate comparison of the performance of the models can only be done after taking into consideration the datasets they have used. The deep networks have not shown the same amount of improvement over handcrafted feature techniques in video processing as they had in image processing. Some of the state-of-the-art handcrafted approaches are on par with deep approaches. Handcrafted approaches like ‘dense trajectory’ [71,74] have provided better results than some of the deep approaches, such as in [96,102], as is evident from Table 3. A possible reason might be that the available labeled images datasets are much larger than the labeled video datasets. Another consideration is that the architecture of CNN, which is the most widely used for image classification, is inherently better suited for treating images as independent elements and does not have the ability to directly incorporate time information spanning over multiple sequences. For this purpose, we have seen the use of RNNs and LSTMs to be able to add sequence-related information into models [95,100,127].

Even though much of the research has shifted towards adapting deep networks for action recognition tasks, deep networks have not completely replaced the traditional handcrafted approaches. A few approaches have focused on getting the benefit of both techniques, i.e., handcrafted features and learned representations, by employing the concept of ‘transfer learning’ as in the works of [96]. Dense trajectory solutions [74] are an example of how well the handcrafted approaches can perform on smaller, but challenging datasets, where deep approaches are limited by the size and quality of datasets. A majority of learning-based approaches to action recognition either directly apply CNN to videos or employ a variation of it to learn features.

In deep networks, spatio-temporal networks and two-stream networks have given better performance than their counterparts. Both of these solutions build on the traditional 3D convolutional architecture by using 3D filters. To obtain temporal information, dedicated streams that use optical flow trajectories have been used, which have been very successful on datasets [102,107], but have the problem of over-fitting. The flow trajectories trained on one set cannot be effective to the same degree on all sets. Deeper networks also perform better than shallower ones [107], but training deeper networks requires better augmented data available in larger amounts other than the severe resource constraint they apply in terms of the number of parameters to tune.

One area that will require further exploration in the future is the idea of pairing video recognition architectures with image recognition ones [20]. Furthermore, multi-stream networks that carry forward more context information should be explored in conjunction with spatial feature recognizers. LSTMs have also shown promising results [95], and their recurrent nature may support the transfer of more complex context information. It is yet to be seen how unsupervised and semi-supervised techniques can be used in conjunction with supervised ones to improve the overall results.

## 7. Conclusions

The ability of machines to understand images and scenes has driven many researchers to find incredible solutions by machine learning. We saw that from simple techniques like MLD (Moving Light Displays) to deep approaches, over time, many solutions have been proposed to find a solution to this problem. Techniques that were used for image understanding have been extended to work for action recognition through videos, as well, with considerable success. However, the problem of action recognition through videos is far more complicated than image analysis. A discussion has been presented to find the techniques that have been used over time and to highlight the most successful ones, in the two dominant categories of ‘deep learning’ approaches and ‘non- deep learning’ approaches, while finding the direction for future research.

## Figures and Tables

**Table 1:** Surveys and studies on action and motion analysis.

Survey	Scope
Poppe [4]	Handcrafted action features and classification models
Aggarwal and Ryoo [15]	Individual and group activity analysis
Turaga et al. [1]	Human actions, complex activities
Moeslund et al. [2]	Human action analysis
Poppe [3]	Human action recognition
Cheng et al. [16]	Handcrafted models
Aggarwal and Cai [17]	Human action analysis
Gavrila [18]	Human body and hands tracking-based motion analysis
Yilmaz et al. [22]	Object detection and tracking
Zhan et al. [23]	Surveillance and crowd analysis
Weinland et al. [24]	Action recognition
Aggarwal [25]	Motion analysis fundamentals
Chaaraoui et al. [26]	Human behavior analysis and understanding
Metaxas and Zhang [27]	Human gestures to group activities
Vishwakarma and Agrawal [28]	Activity recognition and monitoring
Cedras and Shah [29]	Motion-based recognition approaches

**Table 2:** Datasets used for action recognition in increasing order of complexity.

Dataset	Type	No. of Videos	No. of Classes	No. of Subjects
KTH [119]	Indoor/Outdoor	600	6	25
Weizmann [42]	Outdoor	90	10	9
CAVIAR [120]	Indoor/Outdoor	80	9	numerous
UCFSports [121]	Television sports	150	10	numerous
UCF-50 [122]	YouTube videos	-	50	numerous
UCF-101 [123]	YouTube videos	13,320	101	numerous
Sports-1 M [96]	YouTube sports	1,133,158	487	numerous
Hollywood2 [124]	Clips from Hollywood movies	1707	12	numerous
HMDB-51 [125]	YouTube, movies	7000	51	numerous

**Table 3:** Comparison of various action recognition techniques.

Paper	Year	Technique	UCF-101	HMDB-51	Others
Handcrafted Features					
Wang et al. [71]	2011	Dense Trajectory			UCF Sports 88.2
Wang et al. [74]	2013	Dense Trajectory			UCF-50 91.2
Learned Models					
Ji et al. [93]	2013	3D Convolution			KTH 90.2
Tran et al. [97]	2015	C3D generic descriptor	90.4		
Karpathy et al. [96]	2014	Slow fusion			Sports-1 80.2
Sun et al. [98]	2015	Factorized spatiotemporal CovNets	88.1	59.1	
Wang et al. [107]	2015	Two-stream	89.3		
Ng et al. [95]	2015	Conv Pooling		88.2	Sports-1 73.1
Ng et al. [95]	2015	LSTM		88.6	
Donahue et al. [100]	2015	LRCN	82		
Jiang et al. [73]	2012	Trajectories	78.5	48.4	
Varol et al. [94]	2017	Long-term temporal convolutions	91.7	64.8	
Li et al. [126]	2016	VLAD	92.2		
Hybrid Models					
Simonyan and Zisserman [102]	2014	Two-stream CNN	88.0	59.4	
Feichtenhofer et al. [106]	2016	ResNet	93.5	69.2	
Wang et al. [107]	2015	Trajectory pooling + Fisher vector	91.5	65.9	
Lev et al. [127]	2016	RNN Fisher vector	94.08	67.71	
Bilen et al. [128]	2016	Dynamic Image network	89.1	65.2	
Wu et al. [129]	2015	Adaptive multi-stream fusion	92.6		
Deep Generative Models					
Srivastava et al. [109]	2015	LSTM autoencoder	75.8	44.1	
Mathieu [117]	2015	Adversarial network	≈90		
